# *The Ins and Outs of Cathepsins*: Physiological Function and Role in Disease Management

**DOI:** 10.3390/cells9071679

**Published:** 2020-07-13

**Authors:** Tulasi Yadati, Tom Houben, Albert Bitorina, Ronit Shiri-Sverdlov

**Affiliations:** School of Nutrition & Translational Research Maastricht (NUTRIM), Department of Molecular Genetics, Maastricht University, Universiteitssingel 50, 6229 ER Maastricht, The Netherlands; t.yadati@maastrichtuniversity.nl (T.Y.); tom.houben@maastrichtuniversity.nl (T.H.); a.bitorina@maastrichtuniversity.nl (A.B.)

**Keywords:** Lysosomes, cathepsins, translocation, site-specific functions, targeted-drug delivery

## Abstract

Cathepsins are the most abundant lysosomal proteases that are mainly found in acidic endo/lysosomal compartments where they play a vital role in intracellular protein degradation, energy metabolism, and immune responses among a host of other functions. The discovery that cathepsins are secreted and remain functionally active outside of the lysosome has caused a paradigm shift. Contemporary research has unraveled many versatile functions of cathepsins in extralysosomal locations including cytosol and extracellular space. Nevertheless, extracellular cathepsins are majorly upregulated in pathological states and are implicated in a wide range of diseases including cancer and cardiovascular diseases. Taking advantage of the differential expression of the cathepsins during pathological conditions, much research is focused on using cathepsins as diagnostic markers and therapeutic targets. A tailored therapeutic approach using selective cathepsin inhibitors is constantly emerging to be safe and efficient. Moreover, recent development of proteomic-based approaches for the identification of novel physiological substrates offers a major opportunity to understand the mechanism of cathepsin action. In this review, we summarize the available evidence regarding the role of cathepsins in health and disease, discuss their potential as biomarkers of disease progression, and shed light on the potential of extracellular cathepsin inhibitors as safe therapeutic tools.

## 1. Introduction

Lysosomes are intracellular membrane-bound organelles characterized by an acidic interior and harbor a variety of hydrolytic enzymes including lipases, proteases and glycosidases that participate in cellular catabolism [1,2]. The functions of most of these enzymes require an acidic lumen, which is maintained by the vacuolar H+ ATPase (V-ATPase), an ATP-driven proton pump located on the lysosomal transmembrane. Lysosomes can fuse with endosomes, phagosomes, autophagosomes and break down both endogenous and exogenous cargo consisting of various biomolecules such as lipids, proteins, polysaccharides, and certain pathogens. Additionally, lysosomes play critical roles in some of the most vital processes such as metabolic signaling, repair of the plasma membrane and nutrient sensing [3,4,5]. Lysosomes perform this multitude of coordinated events with the help of their enzymes.

Among the variety of enzymes that lysosomes harbor, cathepsins are a family of lysosomal proteases with an astonishingly broad spectrum of functions. Cathepsins help in intracellular house-keeping where they for example, participate in the antigen processing during immune responses and degrade several proteases and chemokines to maintain cellular homeostasis (reviewed in [6,7]). Mammalian proteases have been classified into 5 different families namely metallo, serine, threonine, aspartic, and cysteine proteases based on the type of amino acid at the active site. All cathepsins fall into three different protease families viz; serine proteases (cathepsins A and G), aspartic proteases (cathepsin D and E) and eleven cysteine cathepsins (cathepsins B, C, F, H, K, L, O, S, V, X, and W) [6,8,9]. Serine proteases constitute up to 31% of total proteases expressed in human body, while cysteine and aspartic proteases make up for 25% and 4% of the total protease population respectively [6,10]. Cathepsins show highest activity in the low pH environment of lysosomes. However, certain cathepsins are also found to be active outside of their optimal pH range of 5 [6,11]. The pH optimum of cathepsin S was found to be 6.5 [12,13]. While cathepsin D shows optimal activity at pH 4, its activity was detected even at pH of 7.4 (although at reduced kinetic rates) [14]. Moreover, cathepsin K and H displayed stable activity at pH 7 [15] indicating their wide range of proteolytic activity. Due to the retained activity far outside the optimal pH range cathepsins have been identified with specific proteolytic functions even outside of the endo/lysosomal system [16,17].

Although cathepsins exhibit some level of similarity in their proteolytic functions in physiological processes, consequences of cathepsin dysfunction are very diverse in terms of clinical symptoms. Deregulated cathepsin synthesis and activity has been associated with several diseases including the metabolic syndrome, cancer and inflammatory neurological diseases (reviewed in [18]). Cathepsins are known to activate and/or degrade several important neuronal proteins, and thus have important roles in neurodegenerative disorders (reviewed in [19,20]). For example, cathepsin D plays an important role in neuronal cell homeostasis whose dysfunction leads to impaired proteolysis of target proteins such as huntingtin, α-synuclein, tau, lipofuscin, apoE resulting in Huntington’s and Parkinson’s amongst other neurological disorders [20]. In addition to cathepsin D, several cathepsins are associated with inflammatory neurological diseases including Niemann–Pick type C (NPC) disease, neuronal ceroid lipofuscinosis (NCL) and Alzheimer’s (reviewed in [21,22,23]). Cardiovascular disorders such as cardiomyopathy, hypertension, myocardial infarction, atherosclerosis and aortic aneurysms are characterized by extensive extracellular matrix (ECM) degradation and remodeling, one of prime processes mediated by cathepsins (reviewed in [24]). Similarly, in cancer, tumors metastasize by ECM degradation and cathepsins are known to breakdown the constituents of ECM, epithelial membrane and cell–cell junctions facilitating cancer cell migration. Further, cathepsins are involved in growth, invasion, angiogenesis, and therapeutic resistance associated with tumors [25]. Obesity and diabetes are most common metabolic disorders and cathepsins L, S, and K are found to have potential roles in these pathologies (reviewed in [26]). Thus, the range and complexity of biological activities reliant on cathepsins make them a common point of interest in diverse diseases. Importantly, owing to their differential function during physiological and pathological conditions, cathepsins are considered as highly relevant targets for therapeutic intervention in this range of diseases.

This review is divided into four parts. In the first part, we provide a basic overview of cathepsin expression and function within the endo/lysosomal compartments. In the next two parts, we emphasize on the mechanisms leading to the translocation of cathepsins into the cytosol and extracellular milieu respectively. Finally, this review further highlights the prospect of using highly specific, targeted cathepsin inhibitors for clinical implementation in various diseases.

## 2. Cathepsins in Lysosomes

Cathepsins exhibit similarities in their cellular localization and biosynthesis with some differences in their expression pattern. Of all the lysosomal proteases, cathepsins L, B, and D are the most abundant with their lysosomal concentrations equivalent to 1 mM [27]. Cathepsins B, H, L, C, X, V, and O are ubiquitously expressed while cathepsins K, S, E, and W show cell or tissue-specific expression. Cathepsin K is expressed in the osteoclasts (multinucleated cells of bone) and in epithelial cells. Cathepsins S, E, and W are mainly expressed in immune cells.

### 2.1. Regulation

Cathepsins undergo transcriptional, translational, post-translational and epigenetic regulation. At transcriptional level, except for cathepsin D all other cathepsins show TATA-independent transcription initiation which defines the transcription initiation site. Cathepsin L, K, C, and B are known to require various transcription factors such as nuclear factor (NF-Y), specificity proteins, Sp1, Sp3 and erythroblast transformation-specific (Ets) family factors for transcription initiation and regulation [28,29]. Cathepsin D is known to be transcriptionally regulated by Peroxisome Proliferator-Activated Receptor γ in dendritic cells [30] and by estrogen in breast cancer cells [31]. Transcript variants for cathepsin B and L have been reported as a result of alternative splicing. These transcripts differ in their mRNA stability and thus are known to be accumulated during tumors [32]. Cathepsin L is found to be translationally regulated by internal ribosomal entry site (IRES) [33]. Cysteine cathepsins are known to also contain CpG islands in their promoter region and thus are epigenetically regulated by methylation [25]. Additionally, cathepsin enzyme activity is regulated by pH and endogenous protein inhibitors such as stefins, cystatins, and kiniogens [8].

### 2.2. Synthesis

The primary structure of all cathepsins consists of a signal peptide, a propeptide, and a catalytically active mature functional enzyme. Cathepsins are synthesized as preprocathepsins in the endoplasmic reticulum (ER). A N- terminal signal peptide of 20–25 amino acids directs preprocathepsins into the ER lumen where they are cleaved co-translationally by a signal peptidase generating procathepsins, the less active zymogen forms. Simultaneously, N-linked glycosylation occurs within the ER, producing high levels of mannose in the procathepsins [34]. Procathepsins later travel through the Golgi stocks where the mannose residues are modified to mannose-6-phosphate moieties (M6P). The M6P-tagged procathepsins are recognized and bound by M6P receptors in the trans-Golgi network (TGN) and are either directly sorted to the endo/lysosomes or first pass the plasma membrane from where they enter the endo/lysosomes in an indirect manner. Both direct and indirect routes rely on clathrin-coated vesicles to carry the cathepsins from the TGN or plasma membrane to the endo/lysosomes. Once the cathepsins are trafficked to endo/lysosomes, free M6P receptor is transported back to the TGN [35]. Inside lysosomes cleavage of propeptide converts procathepsins to mature active cathepsins. This activation occurs via different modes such as auto-activation or trans-activation or both. During the auto activation the pH inside the lysosomes enables the cleavage of propeptide by the catalytic site of the same enzyme, while trans-activation requires help of other proteases. For instance, cathepsin B, H, L, S, and K are activated by auto-activation, while cathepsins C and X need cathepsins L and S for their activation [36,37]. Cathepsin D is found to be processed by partial auto-activation and requires cathepsin B and L for further maturation [38]. Further, procathepsin D is known to undergo a two-step maturation process in the endo/lysosomal compartments: in the first step, propeptide is partially cleaved to generate an active single-chain intermediate which upon reaching lysosome undergoes second processing in the region of T^155^–K^173^ to yield a double-chain mature cathepsin D [39].

#### M6P-Independent Sorting

While most of the lysosomal enzymes are dependent on M6P for their transport, several studies have reported the existence of M6P-independent transport routes of cathepsins to reach endo/lysosomes with the aid of alternative receptors. One such alternative receptor protein is sortilin, a trans-membrane Golgi protein, which was shown to be involved in the sorting of cathepsin H and cathepsin D in COS-7 cells [40]. In mouse embryonic fibroblasts cathepsin B and D are known to be captured by the membrane proteins LRP1 (low-density lipoprotein receptor-related protein 1) and LDLR (low-density lipoprotein receptor) [41,42]. While procathepsin D is known to complex with sphingolipid activator precursor protein prosaposin, procathepsin B is known to rely on its membrane association to enter lysosomes independent of M6P route [43,44,45]. Recently, type 1 transmembrane protein SEZ6L2 was linked with sorting of cathepsin D in neurons [46]. Thus, cell-type, post-translational processing, and modifications play an important role in the sorting of cathepsins towards lysosomes.

### 2.3. Physiological Functions of Cathepsins in the Endo/Lysosomes

Cathepsins carry out many proteolytic events in the compartments of the endocytic pathway thus contributing to the protein turn-over and normal metabolism of the cell. The pH inside the endo/lysosomal compartment favors cathepsin activity while it induces conformational changes in the substrates leading to their cleavage by cathepsins, and thereby helping cathepsins to successfully degrade the cargo transported to the endo/lysosomes [9,47].

#### 2.3.1. Immune Responses

Cathepsins display significant roles predominantly in the endosomes of immune cells. They are known to participate both in the innate and adaptive immune responses. During the innate immune responses, lysosomal cathepsins have been shown to cleave the ectodomains of Toll-like receptors (TLRs) 7 and 9 that are expressed on endo/lysosomal membranes, where TLRs recognize nucleic acids of phagocytosed microbes. The processed forms of TLR 7 and 9 then recruit the adaptor protein MyD88 leading to activation of TLR signaling pathways [48]. During adaptive immune responses, T lymphocytes recognize processed antigens on the surface of antigen-presenting cells (APCs) that are bound to major histocompatibility complex (MHC) molecules. While MHC class I molecules present processed antigenic peptides that are derived from the cytosol, MHC class II molecules present peptides derived from the endo/lysosomal compartment. Further, MHC class II molecules require an invariant chain (Ii), a glycoprotein necessary for their folding and assembly. Various cathepsins are found to be involved in the (I) proteolytic processing of antigens into short peptides and (II) degradation of the invariant chain thus facilitating adaptive immune responses [49,50]. However, Deussing et al. [51] demonstrated that cathepsins B and D are non-essential in MHC II mediated antigen presentation.

#### 2.3.2. Autophagy

Cathepsins participate in autophagy, an essential catabolic process which delivers cytosolic constituents to lysosomes for their subsequent degradation in order to maintain cell homeostasis [52]. As demonstrated by Dennemarker et al. [53], cathepsin L deficient primary mouse embryonic fibroblasts showed normal initiation of the autophagy process, autophagosome formation as well as autophagosome–lysosome fusion but impaired degradation of autolysosomal content. Despite this impairment of autolysosomal turnover due to the lack of cathepsin L, the viability of the cells was not affected. This implies that autolysosomal degradation is not solely dependent on cathepsin L and is likely compensated by cathepsin D as evidenced by the increased levels of cathepsin D in cathepsin L knock out cells. In addition, cathepsins also regulate lysosome and autophagosome populations. Cathepsin B is known to degrade the calcium channel MCOLN1/TRPML1 in the lysosomes, leading to the suppression of transcription factor TFEB and thus inhibiting the expression of autophagy-related proteins. This response is known to keep in check the lysosomal biogenesis and population of cellular autophagosomes [54]. Cathepsin S is required for the fusion processes of autophagosomes and lysosomes and its deficiency results in increased number of autophagosomes. Moreover, cathepsin S-mediated autophagic flux is known to induce M2-type polarization of tumor associated macrophages, which would then contribute to tumor [55].

#### 2.3.3. Growth and Development Related Functions

Cathepsins regulate growth and development related processes by processing various hormones and growth factors. For instance, cathepsin B degrades a variety of substrates including insulin-like growth factor-1 (IGF-I), glucagon, pituitary hormone, thyroglobulin [56,57]. Additionally, cathepsin B in the adipocytes is known to cleave perilipin 1 (PLIN1), a lipid droplet-associated protein leading to increased lipolysis in obese adipose tissue [58]. Further, the proteolytic events regulated by cathepsins are critical in the control of biological processes including ovulation, neuronal development and fertilization. For instance, cathepsin L is one of the key proteases upregulated in granulosa cells of ovulatory follicles mediating follicular rupture during ovulation [59]. Cathepsin D is known to process vitellogenin and together with B and L, is involved in oogenesis in lower vertebrates. Cathepsins are extensively involved in embryo development. Relevantly, knock down of cathepsin D led to tissue defects including eye, skin and swim bladder in zebra fish [60] and caused intestinal mucosa damage, atrophy of myelin sheath and early death in mice [61]. Similarly, mice deficient in cathepsins B and L developed atrophy in the cerebral and cerebellar regions of the brain, suggesting their necessity for neuronal development [62].

### 2.4. Pathological Role of Cathepsins in the Endo/Lysosomes

Inactivation or loss-of-function of cathepsins results in inappropriate degradation and abnormal accumulation of substrates in lysosomes leading to various diseases including lysosomal storage disorders (LSD). Further, defective proteolytic events by cathepsins can lead to several LSDs, namely, galactosialidosis [63] neuronal ceroid lipofuscinosis (CLN) type 10 and 13 [63,64], Papillon–Lefèvre syndrome [65], pycnodysostosis [66], and Alzheimer’s disease [21]. Further, given the important role of cathepsins in autophagy, impaired proteolysis by cathepsins can lead to massive accumulation of autophagosomes which eventually can cause pathologies including cellular senescence, inflammasome activation [67] and Saposin (Sap) C deficiency, a rare variant form of Gaucher disease [68]. Impaired activity of Cathepsin D and L would result in accumulation of α-synuclein amyloid fibrils in the brain tissue leading to synucleinopathies [69]. Alterations or loss of functions in the cathepsin activity are known to have adverse effects on reproduction and fertility [70]. Thus, consequences of cathepsin inactivation translate to a wide variety of clinical complications. Detailed functions of individual cathepsins and the associated pathologies are mentioned in the Table 1.

## 3. Cathepsins in Cytosol

As described in the previous section, proteolytic functions of cathepsins mainly occur in the endo/lysosomal compartments. However, most of the cathepsins are released into the extralysosomal locations such as cytosol, nucleus, and mitochondria where they perform crucial tasks. In the current section, we describe the mechanism of cathepsin translocation into different regions of the cell and their respective functions.

### 3.1. Mechanism of Translocation

Perhaps one of the most simplistic mechanisms though which cathepsins are translocated to cellular compartments other than lysosomes is by leaking outside of lysosomes. Lysosomes are surrounded by a limiting membrane, which is a phospholipid bilayer that is characterized by variety of integral membrane proteins, which together protect the organelle from the degradative enzymes [79]. Damage to the lysosomal membrane components or disturbances in membrane fluidity and structure can influence lysosomal stability and subsequently lead to rupture of lysosomes, a process called lysosomal membrane permeabilization (LMP). LMP is induced by a plethora of stimuli including oxidative stress, lysosomotropic agents, as well as some endogenous cell death effectors. While partial permeabilization of the lysosomal membrane induces apoptosis (programmed cell death), complete lysosomal rupture leads to necrosis (reviewed in [80]).

#### 3.1.1. Oxidative Stress-Induced LMP

Lysosomal destabilization is recognized feature during oxidative stress-mediated cell damage. The initial burst of reactive oxygen species (ROS) may be generated by different triggers inside or outside of the lysosomes. For example, ceramide accumulation or ingestion of heavy metals such as silica or asbestos is known to increase NAPDH oxidase in the lysosomes to generate ROS. The generated free radicals interact with free intralysosomal iron forming highly reactive hydroxyl radicals in a Fenton-type reaction [80]. Such hydroxyl radicals induce LMP by causing lipid peroxidation of lysosomal membranes thereby forming lipofuscins and further damaging lysosomal membrane proteins (reviewed in [81]). An oxidative burst elicited by interferon-γ was reported to induce LMP and cathepsin release [82]. Further, ROS is known to modify lipids and lysosomal trapping of oxLDL has the potential to damage and disrupt the lysosomal membrane leading to the secretion of cathepsins [83,84]. Additionally, ROS is found to be generated outside of the lysosomes by destabilized mitochondria, inhalation of exogenous pollutants and ionizing radiation leading to LMP (reviewed in [85,86]).

#### 3.1.2. Lysosomotropic Agents

Lysosome-targeting agents, referred to as lysosomotropic agents and lipid detergents cause LMP either by direct membrane lysis or by osmotic lysis of lysosomal membrane. Examples of agents that cause direct membrane lysis include O-methyl-serine dodecylamide hydrochloride (MSDH), N-dodecylimidazole, n-β-naphthylamide, and endogenous compounds such as lipofuscin. Due to their amphiphilic nature, these agents partition between the water phase and the phospholipid bilayer of lysosomal membrane. Further, accumulation of the lysosomotropic agents and molecules leads to thinning of the lysosomal bilayer and subsequent solubilization of lysosomal membrane (LM) (reviewed in [87]). Lysosomotropic amines, such as chloroquine or ammonium chloride and peptides such as (LeuLeu)nOMe are known to change the permeability of LMs and thus leading to increased influx of solutes into lysosomes and eventually leading to osmotic lysis [88]. Loss of membrane cholesterol or modification of membrane lipids by lipases such as sphingomyelinase and ceramidase is known to induce LMP by increasing the lysosomal permeability for potassium ions and protons and subsequent osmotic lysis [89,90]. Thus, various molecules can cause LMP, leading to leakage of cathepsins into the cytosol.

### 3.2. Transport of Cathepsins to Different Regions of Cytosol

Besides LMP, other mechanisms such as alternative translation or exon skipping can lead to extra lysosomal translocation of cathepsins. Translation initiation at a different start site produces cathepsins which are devoid of signal peptide, while exon skipping generates truncated cathepsins with modified signal sequences thus misrouting cathepsins from their biosynthetic pathway to locations such as cytosol, nucleus, and mitochondria. For instance, translational initiation at downstream AUG site is known to localize cathepsin L in nucleus [91] and alternative splicing in cathepsin B mRNA with missing exons at 2 and 3 is known to direct cathepsin B to mitochondria [92], Another report by Bestvater et al. [93] proposed an alternative targeting signal besides the usual N-terminal signal peptide for cathepsin B namely, a signal patch within its heavy chain domain that facilitates its nuclear import.

### 3.3. Physiological Functions of Cathepsins in the Cytosol

Although cathepsins do not retain optimal activity at the neutral pH of the cytosol, their proteolytic activity is known to be preserved by substrate binding and acidification of the cytosol observed under certain conditions like apoptosis and cathepsins in the cytosol are known to mediate key physiological processes as described below.

#### 3.3.1. Apoptosis and Necroptosis

Apoptosis is a highly regulated, fundamental physiological process of cell death responsible for removal of damaged/aging cells by activation of caspase family of proteins. Apoptosis involves two different pathways; intrinsic pathway, also known as the mitochondria pathway, or the extrinsic pathway involving death ligands (reviewed in [94]). The mitochondria pathway is regulated by B-cell lymphoma-2 (Bcl-2) family proteins. Bcl-2 family consists of both anti-apoptotic members such as Bcl-2 and Bcl-xL and pro-apoptotic proteins including Bax, Bak, and Bid [95]. By genetic modification or use of pharmacological inhibitors, several studies have established that cathepsins in the cytosol play an important role in apoptosis, specifically by activating other apoptotic proteases [96]. Cytosolic cathepsins B, D, and L are implicated in the degradation of Bid, resulting in its activation and translocation to mitochondria. This translocation leads to cytochrome C release from mitochondria followed by caspase activation, and thus initiating apoptotic cell death. Simultaneously, cathepsins are involved in the degradation of anti-apoptotic proteins Bcl-2, Bcl-xL, Mcl-1, and XIAP (X-linked inhibitor of apoptosis), promoting apoptosis [97]. Additionally, in T cells, cathepsin-D-mediated apoptosis involves the activation of Bax, and the release of apoptosis-inducing factor (AIF) and cystatin c. This process has been shown to be independent of Bid cleavage and initiates apoptosis by directly activating the initiator caspase-8 [98,99,100].

Recent studies have shown that cathepsins regulate programmed necrosis termed necroptosis. Necroptosis is initiated by various stimuli and requires the kinase activity of receptor-interacting serine/threonine kinase1 (Rip1). In macrophages, it has been reported that cathepsins B and S are known to cleave receptor-interacting protein 1 (Rip1) kinase and thus limit macrophage necroptosis [101].

#### 3.3.2. Inflammation

Cytosolic cathepsins are shown to be involved in mediating inflammatory responses via activation of inflammasomes. The NLRP3 inflammasome is a multi-protein complex which is activated upon bacterial infections, LMP or cellular damage. Upon activation, pro-caspase1 is converted to active caspase1 by autocatalysis. Active caspase1 then proceeds to cleave the cytokine precursors pro IL-1β and IL-18 into their mature secreted forms (reviewed in [102]). Gene knockout and siRNA knockdown of cathepsins B, C, S, L, and Z resulted in a suppression of IL-1β secretion [103,104,105]. However, cathepsins are not shown to directly cleave either caspase-1 or IL-1β suggesting that they operate upstream of inflammasome activation [103]. Additionally, Cathepsins Z and S are known to compensate for the activity of cathepsins B, C, and L in LMP-mediated inflammasome activation by unknown mechanisms [106].

#### 3.3.3. Functions of Nuclear Cathepsins

Cathepsins in the nucleus are known to process transcription factors that control cell cycle progression thus facilitating cell proliferation and differentiation (reviewed in [107]. The CDP/Cux/Cut transcription factors are a group of highly conserved proteins in higher eukaryotes that are involved in cell cycle proliferation, particularly in the transition from G1 to S phase. Cathepsin L in the nucleus is known to cleave the CDP/Cux, which accelerates progression into S phase of the cell cycle [108]. Nuclear cathepsin D promotes cell proliferation by acting as co-factor for Tricho-rhino-phalangeal syndrome Type 1 (TRPS1) transcription factor and is known to enhance mammary gland differentiation by cleaving histone H3 [109]. Nuclear cathepsins are known to regulate transforming growth factor-β (TGF-β) signaling, an important pathway normal growth and tissue development whose mis regulation can lead to carcinogenesis. The downstream effectors of TGF-β signaling, the Smad proteins, are phosphorylated and activated by receptors such as importinβ which mediate pSmad nuclear translocation, where they regulate transcription. Cathepsins B, K, L, and S are known to localize to nuclear membrane where they exert differential effects in the translocation of pSMAD2 and pSMAD3 proteins by modulating importin β expression and thus regulate TGF-β signaling [110]. Physiological functions of cytosolic cathepsins are listed in Table 2.

### 3.4. Pathological Functions of Cathepsins in the Cytosol

Lysosomal disruption and the subsequent release of cathepsins in the cytosol can have detrimental effects. For instance, though apoptosis is a highly regulated fundamental physiological process, there are many pathological conditions including neurodegeneration and ischemia that involve excessive apoptosis in which cytosolic cathepsins are known to play an active role [96,114]. Accordingly, substantial evidence supports the contribution of cathepsins from ruptured lysosomes in the pathology of many neurodegenerative diseases [115]. In contrast to healthy brain, in Alzheimer’s brain, cathepsin D is found in the cytosol and such cytosolic cathepsin D is known to cleave tau protein generating truncated form of tau which in turn forms paired helical filaments leading to neurofibrillary degradation [115]. Cytosolic cathepsin D is also implicated in glaucoma by promoting apoptosis in trabecular mesh work cells, which maintain intraocular pressure of the eye [116]. Furthermore, cytosolic cathepsin D has been proposed as a biomarker of age-related neurodegenerative disorders [117]. In line, it has been found that cathepsin D translocation into the cytosol led to pronounced age-related changes in rats, by increasing the degeneration of neurons [117,118]. NLRP3 inflammasome is essential for defending against bacterial infections and mis regulated NLRP3 inflammasome has been implicated in metabolic inflammatory disorders including type 2 diabetes, atherosclerosis, heart reperfusion injuries, and chronic kidney diseases (reviewed in [119]). Given the known role of cathepsins in modulating inflammasome activation it then also indirectly suggests a pathological relevance of cathepsins in these disorders.

Another cytosolic cathepsin that is associated with disease conditions is cathepsin L. In pathological conditions such as proteinuria and glomerular kidney disease, translocation of cathepsin L to cytosol has been documented [120]. Here, in contrast to its lysosomal counterpart, cytosolic cathepsin L in podocytes (cells in the Bowman’s capsule of the kidneys), is known to degrade cytoskeleton proteins, namely, CD2-associated protein, synaptopodin and dynamin, thus leading to the reorganization of the actin cytoskeleton, proteinuria and subsequent renal failure [121]. Nuclear cathepsin L activity is associated with polycystic kidney disease [121] and alterations in its activity significantly influenced colorectal cancer disease progression [108]. Nuclear cathepsin F activity is found to be correlated with markers of transcriptional regulation in hepatic stellate cells [122]. Mitochondrial procathepsin B is known to induce morphological changes in mitochondrial integrity and thus can lead to cell death [123]. Taken together, cathepsins perform dynamic functions outside of the lysosomes based on their cytosolic location.

## 4. Cathepsins in the Extracellular Space

Numerous lines of evidence demonstrated the presence of cathepsins in the extracellular space. Although the extracellular localization of cathepsins is more commonly observed during pathological conditions, cathepsins are mostly involved in the bone remodeling and plasma membrane repair during physiological conditions.

### 4.1. Mechanism of Translocation

Cathepsins are normally secreted via lysosomal exocytosis or by alternative sorting from Golgi. Usually secretion of cathepsins is often accompanied by their over expression which is commonly observed in cancer and inflammatory conditions [16]. Immune cells are known to secrete high levels of cathepsins. Additionally, osteoclasts, keratinocytes, thyroid cells, and smooth muscle cells also release cathepsins into the extracellular space [124].

#### 4.1.1. Lysosomal Exocytosis of Cathepsins

The secretory pathway of lysosomes, known as lysosomal exocytosis, has been reported in many types of cells and is induced by various stimuli such as wound, cellular stress, cancer, or by signals from cytokines [125]. The induction of exocytosis occurs by the recruitment of lysosomes to the periphery of the cells. This movement of lysosomes is mediated along microtubule track with the help of various kinesins and a multi subunit complex named BLOC-one-related complex (BORC) that promotes kinesin-mediated lysosome movement toward the cell periphery [126]. The movement of lysosomes to the periphery is followed by docking, where the lysosome and plasma membrane are brought into closer contact with the help of soluble N-ethylmaleimide-sensitive factor attachment protein receptor (SNARE) complexes, namely the interaction of v-SNARE (vamp7) on lysosomal membrane with t-SNARE (synaxin 4 and SNAP-23) on the cytoplasmic side of the plasma membrane (PM) [127]. Both lysosomes and PM contain negatively charged lipids in their outer and inner layers, respectively. Hence, cells rely on calcium ions (Ca^2+^) to bridge the opposing charges on the membranes. Further, lysosomal docking and fusion are regulated by transcription factor EB (TFEB). EB modulates lysosomal exocytosis by triggering intracellular Ca^2+^ elevation through the endo/lysosomal cation-channel mucolipin 1 (MCOLN1) [128]. Finally, the fusion of lysosomes with the PM results in the secretion of lysosomal components into the extracellular space. Castro-Gomes et al. [129] elegantly demonstrated that cathepsin B and L are released extracellularly by lysosomal exocytosis and participate in PM repair using in vitro systems.

#### 4.1.2. Alternative Sorting of Cathepsins into Extracellular Space

As described in the Section 2.1 of this review, cathepsins are trafficked to endo/lysosomal compartments of the cell with the help of M6P receptors. Changes in the pH are known to disrupt the recycling of M6P to the Golgi, where its absence leads to the potential re-routing of procathepsins into the extracellular space directly or packaged into the secretory vesicles [37,130]. In contrast, during conditions such as bone resorption, secretion of active form of cathepsins is observed suggesting that the secretion is context dependent. Once secreted, cathepsins can be found attached to the caveolae on the PM, as seen in case of cathepsin B [131] or released in the extracellular space directly or packed inside the secretory vesicles.

### 4.2. Physiological Function of Extracellular Cathepsins

Cathepsins require acidic pH for their optimum activity in contrast to the neutral pH found in extracellular space. However, cathepsins are often secreted as less active procathepsins which are normally stable at neutral pH [132]. For cathepsins secreted in the mature active form, vacuolar H+- ATPases (V-ATPase) are known to provide a local acidic hub in the pericellular to facilitate their prolonged activity in the extracellular space [133]. For instance, in bone lacunae, V-ATPase and the chloride channel create a low pH extracellular environment with the help of H+ and Cl- ion flow [134]. Cathepsins in the extracellular matrix are known to degrade many components of the ECM and thus participate in physiological processes including wound healing [135], bone remodeling [136,137] and processing of prohormones [8].

#### 4.2.1. ECM Degradation

ECM is composed of fibrous proteins such as elastin, collagen, proteoglycans, and fibronectins that form the intricate meshwork for holding the cells embedded within the tissues Further, ECM undergoes constant remodeling and ECM components have several binding sites for growth factors that ultimately control cell adhesion, proliferation, migration, and polarity [138]. ECM conducts mechanical signals that further activate cytoskeletal and intracellular signaling pathways. In healthy tissue, ECM homeostasis is mainly regulated by cathepsins and matrix metalloproteases (reviewed in [139]).

Bone remodeling is a continuous process that involves bone resorption and remodeling performed by specialized cells called osteoclasts. Further, bone and cartilage predominantly contain type 1 collagen. During bone resorption, osteoclasts attach to the surface of bone leading to the creation of an extracellular compartment which is isolated from general extracellular fluid (reviewed in [140]). Numerous studies showed that the active cathepsin K released from osteoclasts into the resorption lacunae is known to degrade type 1 collagen and elastin and thus plays a pivotal role in bone resorption [17,137,139,141]. Moreover, cathepsins are known to degrade two bone ECM proteins, osteocalcin, and osteonectin. While osteocalcin is involved in bone formation and insulin metabolism, osteonectin helps in cell matrix interactions [139,142]. Further, proteoglycans are major constituents of ECM are composed of core protein with covalently attached glycosaminoglycan molecules (GAGs). Various cathepsins are known to cleave the protein core of proteoglycan. For example, cathepsins B and L cleave perlecan, a heparan sulfate proteoglycan, generating LG3 peptide, which is known to have neuroprotective role in brain ischemia [143,144].

#### 4.2.2. Functions of Cathepsins on the Plasma Membrane

Cathepsins on the cell surface are known to be involved in plasma membrane repair. Cathepsin B released from keratinocytes attaches to cells surface where it is known to be involved in keratinocyte migration by degrading components of ECM during wound healing [145]. Another cathepsin that translocate to the plasma membrane is cathepsin X which helps with processes of cell adhesion and signaling [75]. β integrins are cell surface proteins that help in cell adhesion and invasion. Cathepsin X on the plasma membrane is known to cleave regulatory motifs in two different types of β2 integrin receptors. By activating β2 integrin receptor Mac-1, cathepsin X enhances the adhesion of immature dendritic cells to ECM, leading to their activation. Additionally, cathepsin X activates another β2 integrin receptor, LFA-1 enhancing the proliferation of T lymphocytes thus accelerating immune responses [75,146].

#### 4.2.3. Functions of Cathepsins in the Secretory Vesicles

Neurons and endocrine cells carry out cell–cell communication with the help of peptide neurotransmitters. Secretory vesicles in these cells provide regulated secretion of neurotransmitters, which are first synthesized as inactive prohormones. Proteolytic processing of the proproteins or prohormones occurs in the secretory vesicles by cathepsins [147]. Numerous studies have identified cathepsin L and V as key processing enzymes for production of numerous peptide neurotransmitters including neuropeptide Y, enkephalin, cholecystokinin, and dynorphins [25,148]. The environment inside the vesicles is known to have acidic pH which promotes the function of cathepsins to generate active peptides [149,150].

### 4.3. Pathological Functions of Extracellular Cathepsins

While ECM remodeling is an important physiological process, aberrant ECM dynamics can lead to uncontrolled cell proliferation, invasion, and differentiation leading to fatal pathologies including atherosclerosis, cancer, and tissue fibrosis [138]. Extracellular cathepsin activity have been implicated in many of these diseases as elaborated below.

#### 4.3.1. Cancer

Cancer results from abnormal cell proliferation in the body. Increased or abnormal proteolytic activity of cathepsins is known to degrade ECM components facilitating migration and invasion of tumors leading to malignancy (reviewed in [124]). Cathepsins can be secreted from cancer cells and infiltrating immune cells called tumor-associated macrophages (TAMs). Over secretion of cathepsins is associated with their abnormal expression. Several molecular factors that regulate expression of cathepsins in tumor microenvironment are defined. Signal transducer and activator of transcription 3 and 6 (STAT3, STAT6) are known to promote the secretion of procathepsins B, C, S, and Z mainly from macrophages [151]. Collagen 1 is known to induce secretion of procathepsin B by regulating Ets1 transcription factor [152]. Additionally, it has been postulated that cancer microenvironment downregulates M6P receptor mRNA due to overexpression of cathepsins. Due to its weak affinity towards M6P receptor, cathepsin L is known to be directly secreted from TGN before binding to M6P receptor in fibrosarcoma cells [153]. Metastatic tumor cells are known to have defective lysosomal sorting of procathpesin D resulting in its secretion. Further, the acidic tumor microenvironment not only favors the maturation of procathepsins but also promotes their activity [154].

Elevated levels of extracellular cathepsins have been identified in various cancers such as breast, lung, colon, pancreas, skin, prostate, bladder, ovary, and head and neck [25]. In addition to increased levels, increased activity of cathepsins is often associated with activation of tumor-associated cytokines, shedding and cleaving cell–cell adhesion molecules, thereby destroying cell contact and contributing to metastasis. For example, E-cadherin is an important cell adhesion molecule and epithelial tumor suppressor. Extracellular cathepsins B, L and S are known to cleave E-cadherin promoting tumor invasion into surrounding tissues [155]. In contrast, secreted cathepsin D is known to cleave 23 kDa prolactin, a lactogenic hormone produced by pituitary gland to a 16 kDa fragment which has antiangiogenic properties in rat mammary epithelial cells [156] and in bovine corpus luteum but not in humans [157]. Another mechanism through which extracellular cathepsins contribute to cancer progression is by shedding, a process by which proteases release the extracellular domains of cell surface proteins from the cells [158]. Shedding converts membrane-associated proteins into soluble ones and thus reduces their cell surface expression that in turn is a means of regulation for subsequent physiological processes. Apart from several cell adhesion molecules (CAMs) identified as substrates for shedding, cathepsins also target the Ras signaling pathway, a major intracellular signaling pathway in cancer progression. Extracellular cathepsins S and L are known to shed plexins and epidermal growth factor receptor (EGFR), both of which are substrates for Ras pathway activation [16,158]. Similarly, extracellular cathepsin D is known to degrade the ECM proteins thus freeing the embedded growth factors such as fibroblast growth factor. Growth factors are complex polypeptides that have critical roles in basement membrane disruption, cell migration, and tumor metastasis in auto and paracrine manner [154].

#### 4.3.2. Metabolic Disorders

Lipoprotein accumulation and metabolism are important contributors to various diseases including cardiovascular diseases and obesity -associated disorders such as non-alcoholic steatohepatitis (NASH). Extracellular cathepsins are found to be involved in transport, efflux, and processing of lipoprotein molecules or their receptors (reviewed in [159]). For instance, extracellular cathepsins F, K, and S are known to degrade cholesterol acceptors on the cell surface, thereby reducing cholesterol efflux and initiating foam cell formation, a key feature of atherosclerosis [24]. Increased presence of low-density lipoprotein (LDL) is a characteristic of both atherosclerosis and NASH. Extracellular cathepsin D is known to proteolytically modify apolipoprotein B-100 (apoB), component of LDL and subsequently leading to LDL accumulation in arterial intima [160,161]. In agreement, extracellular cathepsin D inhibition is known to reduce hepatic steatosis [162]. Further, LDL accumulation in the arterial intima can lead to its oxidation which enhances inflammatory responses, that in a positive feedback loop promotes further secretion of cathepsins leading to exacerbation of lipid accumulation and inflammation (reviewed in [163]). Similar to extracellular cathepsin D, extracellular cathepsin S is known to promote inflammation by cleaving chemokines such as fractalkine (CX3CL1) that help in leukocyte migration and neuropathic pain [164]. Finally, extracellular cathepsin activity is involved in lung fibrosis, osteoarthritis, osteoporosis and rheumatoid arthritis which are summarized in Table 3.

**Figure 1:**
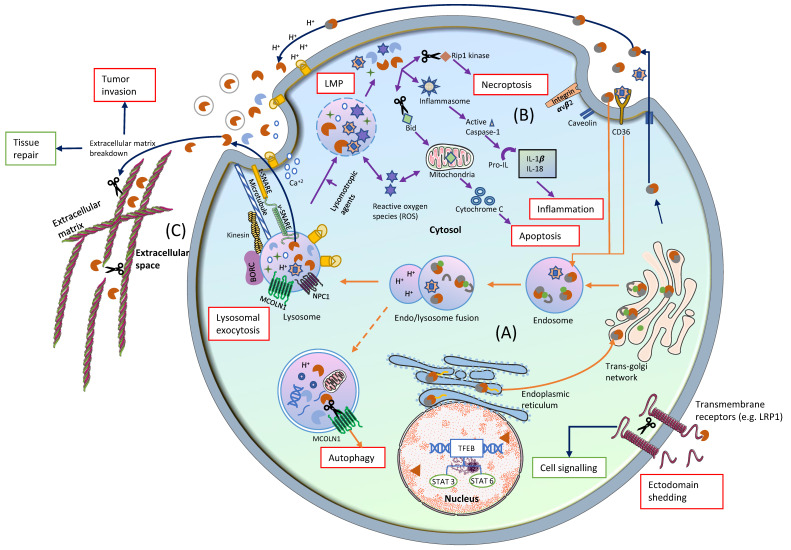

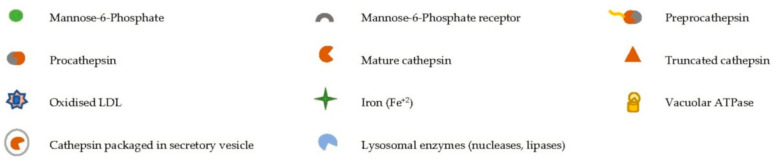
**Site-specific functions of cathepsins**. (**A**) cathepsins in the lysosomes (represented by orange arrows): Cathepsins are synthesized as preprocathepsins in the endoplasmic reticulum and transported to endo/lysosomes via Trans-Golgi network where the acidic pH enables their maturation. Cathepsins in the lysosomes are mostly involved in protein degradation besides participating in autophagy. (**B**) cathepsins in the cytosol (represented by purple arrows): Lysosomotropic agents, ROS or accumulation of modified lipids (oxLDL) leads to lysosomal membrane permeabilization (LMP), releasing cathepsins into the cytosol. Cytosolic cathepsins participate in various activities. For example, cathepsins trigger the inflammasome and promote apoptosis and necroptosis by cleaving various proteins. (**C**) Cathepsins in the extracellular space (represented by blue arrows): Lysosomal exocytosis involves the secretion of lysosomal contents into the extracellular space with the help of several protein-receptor interactions and Ca^+2^ ion gradient. Cathepsins are released in the form of procathepsins or enclosed in the secretory vesicles or as active cathepsins. Secreted cathepsins remain attached to the plasma membrane or are released into the extracellular space. Cathepsins on the plasma membrane cleave proteins like integrins. Secreted cathepsins mainly participate in extracellular matrix degradation and thus help in wound healing. However, excessive ECM cleavage facilitates tumor invasion and promotes cancer. While in the extracellular space cathepsins also shed the ectodomains of transmembrane receptors, leading to either activation or inhibition of cell signaling. ROS: reactive oxygen species; LMP: lysosomal membrane permeabilization; NPC1: Niemann–Pick disease type C1; CD36: cluster of differentiation 36; Figure is created with permission from Servier Medical Art image bank.

The mechanisms involved in the translocation of cathepsins and their respective site-specific functions are illustrated in Figure 1.

## 5. Targeting Cathepsins in Disease Management

Localization of cathepsins (endo/lysosomal/cytosolic/extracellular space) governs several aspects of cathepsin function. A wealth of knowledge has been published on the differential expression and functional profiles of cathepsins in various pathologies making them potential diagnostic biomarkers and most desirable therapeutic targets.

Cathepsin levels and activity have been found to be upregulated in sera and tumors of many cancer patients [176,177]. For instance, expression and activity levels of cathepsins B and L corelated with breast cancer progression [178]. Furthermore, B and L cathepsins also correlated with relapse rate after treatment in primary breast cancers [178]. In cancers concerning colon, lung, brain and head and neck, concentration of cathepsins B and L within tumors correlated with survival probability [179]. Moreover, cathepsin L levels were increased in sera of patients with pancreatic [180] and liver cancers [181]. Serum cathepsin H levels were found to be increased in patients with lung [182], melanoma [183], and colorectal cancers [184]. The findings of these recent studies have alluded to a potential of utilizing cathepsins as biomarkers of cancer though few studies show some discrepancies in the outcomes of cathepsin expression and activity during cancer (reviewed in [185]). In addition to cancer, plasma levels of cathepsin S, K, and L have been proposed as biomarkers in coronary artery disease, aneurysm, adiposity, and peripheral arterial disease [186]. Similarly, plasma cathepsin D levels associated with metabolic alterations in liver during NAFLD [187]. Recent studies suggested that plasma cathepsin D levels correlated with type 2 diabetic patients [188] and moreover plasma cathepsin D activity is suggested as biomarker for hepatic insulin sensitivity [189]. Cathepsins Z and K are proposed diagnostic markers for osteoporosis [190,191]. Another promising development is the use of cathepsins as fluorescent probes in diagnostic non-invasive imaging which has had success in preclinical mouse models [192].

In diseases caused by inactivation or loss-of-function of cathepsins, supplying functional cathepsins could be a valuable means to restore cellular function and ameliorate the disease. For instance, enzyme replacement therapy (ERT) replacing defective lysosomal cathepsin D by recombinant procathepsin D has been proven beneficial for CLN10 [193]. However, due to known associations of excessive extracellular cathepsin D with other pathological conditions highlighted previously in this review caution must be exercised when utilizing this therapeutic approach to avoid overaccumulation of cathepsin D in extra lysosomal locations which could possibly lead to activation of extracellular cathepsin D mediated pathological processes. When implementing the ERT approach for cathepsins, the recombinant cathepsins’ mannose-6-phosphate content must be maximized to ensure their optimal uptake by tissues and to avoid their extra lysosomal accumulation. In addition, conjugating recombinant cathepsins with chaperones might help to diffuse them across cell membranes and reach target tissues including central nervous system (CNS) [194]. Additionally, close monitoring of injected levels of recombinant enzyme in patients might help in preventing any negative effects.

Increased understanding of the structure, differential expression and localization of cathepsins in various pathologies has opened a new avenue for the design of small molecule inhibitors of cathepsins with the hope of producing highly specific, targeted drugs for many diseases. Several small molecule inhibitors of cathepsin S and K are being tested in clinical trials [192]. Additionally, combinatorial therapies involving cathepsin inhibition are gaining more attention. For example, cathepsin inhibitors conjugated with radio/chemotherapy would be a potential anti-cancer treatment [195]. Further, cathepsin K clinical inhibitors for treatment of osteoporosis might have potential to attenuate cancer [196]. Unfortunately, one of the bone-specific cathepsin K inhibitors to complete phase III clinical trials, odanacatib (Merck) had to be discontinued due to risk of stroke [197]. The failure was likely due to the fact that the active-site inhibitor of K also blocked the other essential protease functions of cathepsin K [17]. One of the strategies to alleviate this problem was to identify inhibitors specific to the exosites or allosteric sites, that essentially inhibit the pathological collagenolytic activity only and thus limiting the cytotoxicity [198]. One of such exosite inhibitors of cathepsin K was successfully demonstrated in a mouse model of osteoporosis [199]. However, since not all cathepsin activities are modulated by exosite interactions it presents a limitation for the use of exosite inhibitors in certain cathepsin related pathologies. Other novel ways such as site-specific inhibition of cathepsins would also reduce their off-target limits. For instance, small-molecule inhibitors targeting only the secreted extracellular fraction of cathepsin D and not the lysosomal fraction had beneficial results in a rodent model of NAFLD [162]. Moreover, antibodies against extracellular fraction of cathepsin S efficiently inhibited tumor growth and neovascularization in xenograft tumors [200] and improved chemotherapy efficacy in colorectal carcinomas [201]. There are still a few inhibitors targeting either extracellular cathepsins or their substrates that require further validation in clinical setting [192] as described in the Table 4. Taken together, while evidence that shows a direct link between extracellular cathepsins and various pathologies are few, current studies to date show promising therapeutic result of targeting specifically the extracellular fraction of cathepsins in their respective pathologies. Therefore, further research looking into the specific role of extracellular cathepsins and the therapeutic value of targeting them is recommended.

## 6. Conclusions

In conclusion, cathepsins are a group of enzymes with distinct functions at different locations inside and outside of the cells. While many recent findings helped us to further understand these roles and establish the potential for targeting, many mechanical aspects of cathepsin action are still to be explored. Currently, the extracellular role of cathepsins have gained enormous attention in the biomedical field, establishing them as non-invasive diagnostic markers and pharmacological targets in immune disorders, cancer, osteoarthritis, and metabolic diseases. Many selective cathepsin inhibitors with limited side effects are being developed and have shown success in the preclinical animal models and are awaiting validation in the clinical setting. With the advent of new mass spectrometry technologies and systems biology approaches, the future research should focus to better understand the specific substrates of cathepsins in the physiological and pathological environment. Finally, the future of cathepsins in targeted drug delivery looks more promising than ever.

## Figures and Tables

**Table 1:** Cathepsins in the endo/lysosomal compartment.

Cathepsin	Enzyme Commission Number	Catalytic Type	Function	Pathology	OMIM ID	Reference
Cathepsin A	3.4.16.5	serine	dual function: a. protective:β-galactosidase and neuraminidase b. degradative: bioactive peptides like bradykinin, angiotensin, oxytocin, endothelin 1	hypertension Galactosialidosis	256540	[63]
Cathepsin B	3.4.22.1	cysteine	degrades amyloid-β; activation of pro-hormones and pro-enzymes; trypsin activation; promotes viral entry into cells	Alzheimer’s; gaucher disease acute pancreatitis		[21,68]
Cathepsin C	3.4.14.1	cysteine	inflammatory responses and activation of serine proteases including neutrophil elastase and cathepsin G	Papillon–Lefèvre syndrome Periodontitis	245000	[65]
Cathepsin D	3.4.23.5	aspartic	embryo and neuronal development brain antigen processing of α-Synuclein; tau, amyloid β, apoE; degradation of hormones, proenzymes and growth factors	Alzheimer’s disease; CLN 10 Parkinson’s; Huntington’s	610127	[20,61,64]
Cathepsin E	3.4.23.34	aspartic	carboxypeptidase A and IgE processing	atopic dermatitis		[71]
Cathepsin F	3.4.22.41	cysteine	li chain processing and MHC-II class responses	CLN 13	615362	[63]
Cathepsin G	3.4.21.20	serine	auto antigen processing	auto-immune diseases		[72]
Cathepsin H	3.4.22.16	cysteine	prohormone processing	type 1 diabetes		[9]
Cathepsin K	3.4.22.38	cysteine	TLR signaling; processing of β-endorphin in brain	periodontitis; pycnodysostosis	265800	[73,74]
Cathepsin L	3.4.22.15	cysteine	antigen and li chain processing; prohormone processing; degradation of α-Synuclein, tau; promotes viral entry into cells	Parkinson’s disease; frontotemporal dementia		[19,49]
Cathepsin S	3.4.22.27	cysteine	antigen processing and presentation; li chain processing	auto-immune diseases		[50]
Cathepsin X	3.4.18.1	cysteine	T-cell migration and invasion	-		[75]
Cathepsin O	3.4.22.42	cysteine	-	-		
Cathepsin V	3.4.22.43	cysteine	natural killer cell and CD8+ cytotoxic cell production	thymic pathology		[76]
Cathepsin W	3.4.22.-	cysteine	component of endoplasmic reticulum proteolytic machinery	-		[77]
Cathepsin Z	3.4.18.1	cysteine	intracellular protein turnover	-		[78]

- implies that no function or pathology is yet discovered for the respective cathepsins. CLN stands for ceroid lipofuscinosis, neuronal; OMIM stands for online mendelian inheritance in man.

**Table 2:** Function of cathepsins in the cytosol.

Cathepsin	Extra Lysosomal Location	Function	Reference
Cathepsin B, D and L	cytosol	proteolytic processing of Bid during apoptosis	[97,111]
Cathepsin B, C, L, S and Z	cytosol	NLRP3 inflammasome activation	[103,104,105,106]
Cathepsin B	cytosol	regulation of hepatic lipid metabolism by degrading liver fatty acid binding protein	[112]
Cathepsin L and H	nucleus	cell cycle regulation	[91]
Cathepsin B, K, L and S	nucleus	TGF-β signaling	[110]
Cathepsin B	nucleus	bile-salt induced apoptosis	[113]
Cathepsin A, E, G, S, X, O, V, W, Z *	-	-	-

* Roles for these remaining cathepsins outside of the lysosome have not yet been reported.

**Table 3:** Cathepsins in the extracellular space.

Cathepsin	Substrate	Pathological State	Reference
Cathepsin B, K, and L	proteoglycan	osteoarthritis	[137,165]
Cathepsin B, L, G, and S	fibronectin	cancer and adipogenesis	[166,167,168,169]
Cathepsin B, L, and S	laminin	cancer neovascularization, intestinal trauma	[166,167,168]
Cathepsin K	collagen type I	osteoporosis, rheumatized arthritis, osteoarthritis	[137]
Cathepsin B, K, L, and S	collagen type 2	lung fibrosis, cardiovascular diseases and cancer	[138,139]
Cathepsin B	tenascin	cancer	[170]
Cathepsin B, K, L, and S	aggrecan	osteoarthritis	[136,171]
Cathepsin L, S, and B	plexin	tumorigenesis	[158]
Cathepsin S	fractalkine	neuropathic pain	[164]
Cathepsin D	fibroblast growth factor	breast cancer	[172]
Cathepsin V	elastin	cancer	[173]
Cathepsin X	CXCL-12	-	[174]
Cathepsin W	-	cell-mediated cytotoxicity	[175]
Cathepsin A, C, E, F, O, and Z *	-	-	

* Role of these extracellular cathepsins in pathologies in not known.

**Table 4:** List of available extracellular cathepsin inhibitors.

Inhibitor	Target	Reference
Fsn0503	antibody against extracellular cathepsin S	[200]
Nitroxoline	extracellular cathepsin B	[202]
LNC-NS-629	extracellular cathepsin B	[203]
CTD-002	extracellular cathepsin D	[162]

## Abbreviations

AIF	Apoptosis-inducing factor
ATP	Adenosine triphosphate
Bcl-2	B-cell lymphoma-2
BORC	BLOC-one-related complex
CLN	Ceroid lipofuscinosis, neuronal
CNS	Central nervous system
CX3CL1	C-X3-C Motif chemokine ligand 1
ECM	Extracellular matrix
EGFR	Epidermal growth factor receptor
ER	Endoplasmic reticulum
IGF-I	Insulin-like growth factor-1
IRES	Internal ribosomal entry site
LDL	Low-density lipoprotein
LDLR	Low-density lipoprotein receptor
LFA-1	Lymphocyte function associated antigen-1
LM	Lysosomal membrane
LMP	Lysosomal membrane permeabilization
LRP1	Lipoprotein receptor-related protein 1
LSD	Lysosomal storage disorders
MCOLN1	Mucolipin 1
MHC	Major histocompatibility complex
MSDH	O-methyl-serine dodecylamide hydrochloride
NASH	Non-alcoholic steatohepatitis
NAFLD	Non-alcoholic fatty liver disease
NCL	Neuronal ceroid lipofuscinosis
NF-Y	Nuclear factor
NPC	Niemann–Pick disease type C
OMIM	Online mendelian inheritance in man
oxLDL	oxidized low-density lipoprotein
PM	Plasma membrane
ROS	Reactive oxygen species
TAM	Tumor-associated macrophages
TFEB	Transcription factor EB
TGF	Transforming growth factor
TGN	Trans-Golgi network
TLR	Toll-like receptor
V-ATPase	vacuolar H+ ATPase
XIAP	X-linked inhibitor of apoptosis
